# Impact of Milk Thermization on the Quality Characteristics of P.D.O. “Canestrato Pugliese” Ovine Hard Cheese

**DOI:** 10.3390/foods12051080

**Published:** 2023-03-03

**Authors:** Giuseppe Natrella, Giuseppe Gambacorta, Giacomo Squeo, Michele Faccia

**Affiliations:** Department of Soil, Plant and Food Science, University of Bari, Via Amendola 165/A, 70126 Bari, Italy

**Keywords:** ovine cheese, PDO Canestrato Pugliese, milk thermization, proteolysis, sensory analysis

## Abstract

The use of raw milk is compulsory in the manufacturing process of most of the European protected designation of origin (PDO) cheeses but, for ovine products, it is often responsible for faulty productions. Since pasteurization is hardly compatible with the PDO concept, a milder treatment (thermization) is allowed in some cases. An investigation was undertaken to assess the effect of thermization on the overall quality of Canestrato Pugliese, a PDO ovine hard cheese of Southern Italy that can be manufactured exclusively from raw milk. Three types of cheese were produced using raw, mild-thermized and high-thermized milk inoculated with a thermophilic commercial starter. The results demonstrated that the heat treatment did not cause remarkable differences in the gross composition, but the microbiological profiles had some differences despite the use of the selected starter. The raw milk cheese contained higher levels (0.5–1 log units) of mesophilic lactobacilli, total viables, total coliforms and enterococci with respect to the thermized counterparts, with the high-thermized cheese showing the lowest levels; these microbiological differences fitted well with the higher content and the different High Performance Liquid Chromatography (HPLC) pattern of soluble nitrogen. The sensory analysis revealed that the thermized cheeses lost some typical sensory characteristics, probably as a consequence of the reduced indigenous microbiota populations. It was concluded that milk thermization could be applied to Canestrato Pugliese manufacturing only together with the development and use of an autochthonous starter.

## 1. Introduction

Protected designation of origin (PDO) and protected geographical indication (PGI) are two instruments created by the European Union to protect typical food products of specific geographical areas within the European framework [1]. These products are manufactured in a given area using recognized manufacturing practices that are described in the official production protocol forming an integral part of the law establishing the acknowledgement [2]. For both PDO and PGI products, protocol updating is a laborious process, requiring time and attention. That is problematic because, overtime, some specific technical aspects of the manufacturing process can become incompatible with the changes of the food legislation and the consumers’ expectations. This is the case for the use of unpasteurized milk in cheesemaking, whose safety is highly debated worldwide, as well as its unsuitability for obtaining a constant quality [3]. Even though most PDO cheeses continue to be manufactured from raw milk, the possibility of applying thermal treatments is increasingly being required from the producers [4,5] in order to reduce the risk of defects. Milk pasteurization causes the elimination, along with pathogenic microorganisms, of indigenous microbiota, which make the use of a starter in cheesemaking necessary. In addition to this, other modifications can play a role in cheese quality, such as the denaturation of whey proteins, inactivation/activation of native milk enzymes and changes in the mineral equilibrium. All these modifications, whose intensity depends on the parameters of the heat treatment, often impact proteolysis and lipolysis, leading to a different flavor with respect to the cheese obtained from raw milk [6,7,8,9,10]. For all these reasons, pasteurization is considered a problem for PDO/PGI cheeses, as the modifications of the milk characteristics tend to weaken the linkage with the geographical area. In order to minimize the adverse effects, milk thermization could be an alternative to pasteurization. According to the EU regulations, thermization is a sub-pasteurization treatment, performed under mild conditions (i.e., 57–68 °C for no less than 15 s in a heat exchanger), so as to guarantee the preservation of phosphatase activity (CE Dir 46/92) and, consequently, part of the indigenous microbiota. The treatment always causes the elimination of psychrotrophic bacteria and the reduction of total bacterial count, whereas a marked reduction of *Enterobacteriaceae* and coliforms is obtained only adopting suitable time/temperature combinations [11,12,13]. Of course, for thermization it is also necessary to add a starter before cheesemaking, in order to fortify the indigenous microflora surviving the heat treatment that is not sufficient for carrying out the suitable fermentations. This thermal treatment can also be performed directly in the vat if, as often happens at a small farm level, a heat-exchanger is not available. Besides preserving part of the indigenous microbiota, thermization presents other advantages with respect to pasteurization, such as less damage to the milk thermolabile compounds, the better preservation of coagulability and lower energy consumption for heating and cooling.

The possibility of applying milk thermization before cheesemaking is much more requested by the manufacturers of PDO ovine cheeses than the bovine counterpart, since sheep milk quality is often affected by high microbial load. Italy is the European Union (EU) country with the highest number of PDO ovine cheeses (seventeen), all belonging to the class of semi-hard or hard cheeses. Out of seventeen, eight have received the authorization to apply a thermal treatment to milk, while the remaining nine must be manufactured from raw milk [14], as reported in Table 1.

For PDO/PGI cheeses, the ban on the heat treatment could only be removed by demonstrating that the overall quality does not significantly change and the linkage with the territory is not lost. Unfortunately, information on the effect of thermization on cheese quality is scarce. Xanthopoulos et al. [15] investigated Anevato, a Greek PDO spreadable cheese made from goat milk, and concluded that thermization allowed for the better preservation of part of the milk indigenous microbiota with respect to pasteurization (about one-half log unit of difference for the most important microorganisms groups in terms of colony forming units). However, both heat-treated milk cheeses lacked the characteristic cheesy flavor of raw milk cheese. In cheddar cheese, both pasteurization and thermization (65 °C × 15 s) had no effect on primary proteolysis but reduced the levels of free amino acids and the intensity of lipolysis during ripening [8]. Pirisi et al. [16] investigated the effect of milk thermization on the quality of Fiore Sardo and found that the heat treatment did not significantly influence the composition and the secondary proteolysis, whereas significant differences were observed in the rheological properties and in the lypolitic pattern. Caboni et al. [17] investigated the same cheese and concluded that while the effects of milk thermization on macro-compositional parameters and free fatty acid levels were not evident, strong differences between cheese produced from raw and thermized milk were detectable by using a Gas Chromatography- Mass Spectrometry (GC-MS) metabolomics approach. Unfortunately, the study did not include a sensory evaluation. Finally, a recent work of Dedola et al. [18] proposed the dosage of enzyme α-l-fucosidase as a tool for discriminating Fiore Sardo PDO cheeses obtained from raw or thermized milk, but in this case the sensory characteristics were also not investigated.

Canestrato Pugliese is one of the nine ovine Italian PDO cheeses for which the heat treatment of milk is not allowed. It is a semi-hard to hard cheese made in the Apulia Region (Southern Italy) from milk of sheep of local flocks, whose designation of origin is connected to ancient sheep transhumance. Transhumance is a very old form of pastoralism consisting of the seasonal movement of livestock and herders between higher pastures in the summer, and lower pastures during the winter. The north-west part of Apulia has not too cold winters and presents vast lowland areas with natural pastures; for this reason, it has been the summer destination for sheepherders from the surrounding mountain areas since Roman times [19]. As sheep lactation mostly took place from February to May, the Apulian lowlands were crawling with shepherds making cheeses, the most important of which was a hard type that could be easily stored. It was called Canestrato from the name of the rush basket (“Canestro”) in which it was molded and kept for the first period of ripening that lasted from a few months to about one year. When transhumance ceased to be practiced (1950–1960s) and sheep breeding became sedentary, the cheese continued to be manufactured in dairy industries and farmers cooperatives. The uniqueness linked to history, the particular environment in which the flocks graze, the manufacturing procedure and typical organoleptic characteristics led to the acknowledgement of the designation of origin, which was first obtained in 1985 at the national level and then confirmed in 1996 at the EU level. Unfortunately, the recent changes in both livestock management and consumer habits has caused the total production volume of Canestrato Pugliese to dramatically decline in the last two decades. The main problem caused by the changes in consumer habits was the increased demand of “perfect” food products, which led to the depreciation of non-standardized cheeses which often are those obtained from raw milk. In addition to this, the use of raw sheep milk brings with it a high risk of faulty production that cannot be tolerated by the manufacturers. The consequence of this framework is that the dairy industry has abandoned the production of Canestrato Pugliese, which is currently manufactured only in two small artisanal dairies. The absence of interest from the dairy industry, together with difficulties in finding animal care workers, is continuing to discourage the sheep farmers and, if the situation does not change, the Apulian breeding sheep sector will disappear. The application of milk thermization could increase the interest of dairy enterprises to restart the production of this cheese, but no research has been conducted about its effect on cheese quality. Albenzio et al. [20] investigated the effect of milk pasteurization and of heating the curd in hot whey (80 °C for 30 s) on proteolysis and lipolysis. Both treatments led to lower counts of lactic acid bacteria in cheese and slower proteolysis and lipolysis than the control cheese made from raw milk without any thermal treatment. Unfortunately, the cheeses were not subjected to sensory analysis, even though the authors speculated that it should be expected that cheeses obtained from raw milk reach their optimum sensory quality earlier than those from pasteurized milk. Piombino et al. [21] investigated the sensory characteristics of Canestrato Pugliese made from raw or pasteurized milk, and found important differences, which were confirmed by the gas chromatography-mass-spectrometry-olfactometry analysis of volatile compounds. The aim of the present research was to assess the effect of milk thermization on the chemical, microbiological and sensory characteristics of Canestrato Pugliese cheese, in order to establish the extent by which the heat treatment influences the overall quality.

## 2. Materials and Methods

### 2.1. Cheese Manufacturing

**Figure 1:**
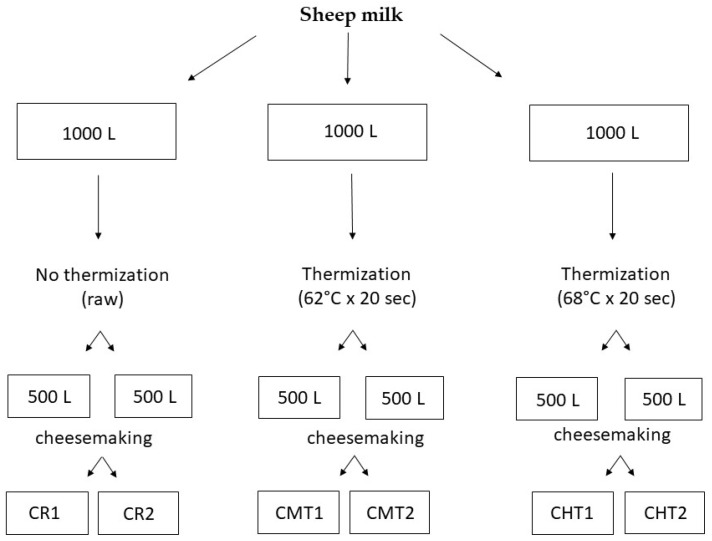
Experimental design applied in the investigation.

**Figure 2:**
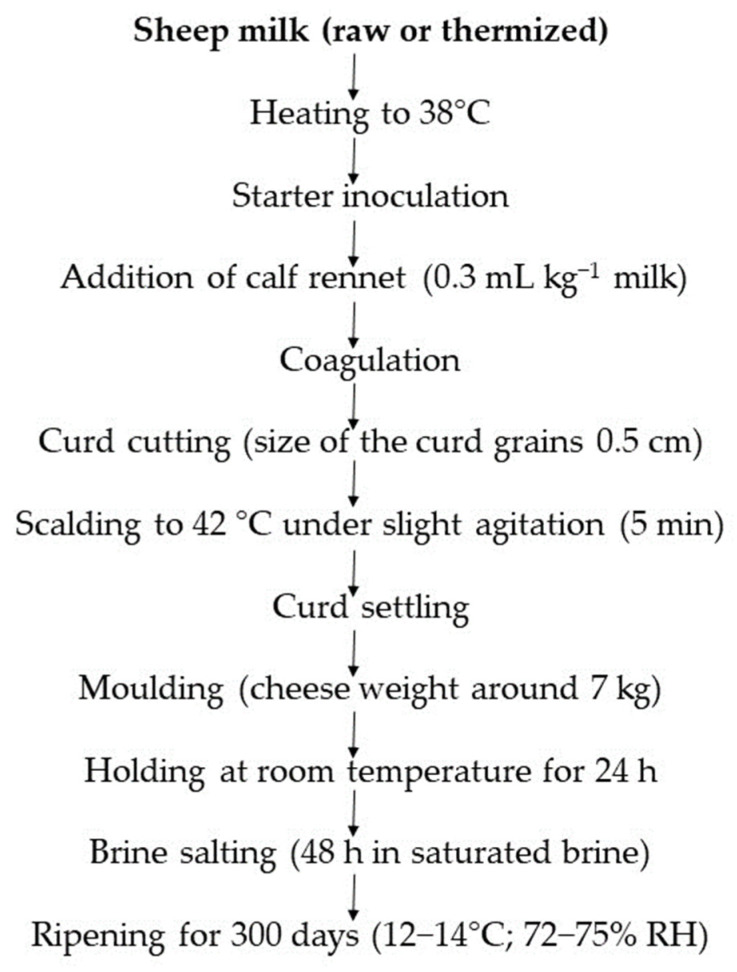
Cheesemaking process applied in the experimentation.

The milk used in the experimentation derived from a batch of ovine bulk milk (about 4000 L) collected in two days from 17 sheep farms located in the province of Bari and Foggia (Apulia region, Italy). From this batch, three 1000 L aliquots were taken, one of which was used raw for preparing the control cheese (CR) while the other two were subjected to thermization in a heat exchanger for preparing thermized milk cheeses. Two different heating conditions were tested: 62 °C × 20 s for preparing mild-thermized cheese (CMT) and 68 °C × 20 s for preparing high-thermized cheese (CHT). Two cheesemaking replicates were performed for each type of milk by applying the official production protocol [22], except for the addition of a selected lactic acid bacteria (LAB) starter (a mixed culture of *Streptococcus thermophilus* and *Lactobacillus delbrueckii* subsp. *bulgaricus*, Sacco Srl, Cadorago, Italy) for reintegrating the microbiota damaged by the heat treatment. The culture was also added to the raw milk for manufacturing the control cheese, in order to normalize the technological conditions. The experimental design is summarized in Figure 1, whereas the cheesemaking protocol adopted is shown in Figure 2.

### 2.2. Chemical Analyses

The milk used in the experimentation was subjected to the analysis of the gross composition by infrared analyzer (Milko-Scan, Foss Electric, Hillerød, Denmark). The cheeses were sampled at 0, 7, 14, 21, 28, 42, 56, 70, 82, 150, 180, 240 and 300 days of ripening. Representative samples were prepared by eliminating the rind (about 0.5 cm) and cutting a triangular wedge weighting 0.5 kg. The wedge was then grated, and, after thorough mixing, portions were taken to perform the analyses. The following determinations were carried out: moisture (oven drying), pH (pH meter equipped with a penetration probe, Hanna Instruments, Woonsocket, RI, USA), NaCl (chloride analyzer, Sherwood Scientific Ltd., Cambridge, UK), fat (Soxhlet method), total nitrogen (Kjeldahl method), water-soluble nitrogen (WSN) according to Kuchroo and Fox [23]. All analyses were carried out in triplicate. Proteolysis was investigated by urea-poly acrylamide gel electrophoresis (PAGE) according to the method of Andrews [24]. The main casein fractions were identified by comparison with a milk sample taken from the vat and with the data from the scientific literature; the protein bands in the gel were quantified by densitometry. Proteolysis was also investigated by Reverse-Phase High Performance Liquid Chromatography (RP-HPLC) analysis of water soluble nitrogen on an Agilent Technologies apparatus with Ultraviolet (UV) detection (Palo Alto, CA, USA), under the operating conditions reported in a previous paper [25].

### 2.3. Microbiological Analyses

Analyses were carried out on raw milk, vat milk (i.e., after starter addition), curd and cheese at 0, 7, 14, 28, 56, 150 and 300 days of ripening. On raw milk, the count of somatic cell and total bacteria was performed by flow cytometry (Fossomatic and Bactoscan, Foss Electric, Denmark). For vat milk, curd and cheese, a 20 mL or 20 g sample was diluted in 180 mL of 2% (*w*/*v*) sodium citrate solution and homogenized in a Stomacher Lab-Blender. Serial dilutions were made in quarter strength Ringer’s solution and plated on specific media for viable counts. The following groups were enumerated: total viable (TV, 32 °C, plate count agar, Oxoid, Basingstoke, UK), total coliforms (TC, 37 °C, VRB agar, Biolife, Milan, Italy); presumptive mesophilic lactococci (MLc, 30 °C, M17 agar supplemented with 0.1% cycloheximide, Merck, Darmstad, Germany); presumptive mesophilic lactobacilli (MLb, 30 °C, MRS agar supplemented with 0.1%, cycloheximide Merck, Darmstad, Germany); presumptive thermophilic lactobacilli (TLb, 45 °C, MRS agar supplemented with 0.1%, cycloheximide Merck, Darmstad, Germany); presumptive thermophilic streptococci (TLs, 45 °C, lactose M17 agar supplemented with 0.1%, cy-cloheximide Merck, Darmstad, Germany); presumptive enterococci (Ec, 37 °C, Slanetz and Bartley agar, Oxoid, Basingstoke, UK); yeasts and molds (Y&M, 25 °C yeast glucose chloramphenicol media, Biolife, Milan, Italy).

### 2.4. Sensory Analysis 

The cheeses were evaluated by a panel composed of 7 trained assessors, selected by following ISO standard 8586-1 and certified by the Italian Association of Cheese Tasters (ONAF, Cuneo, Italy) after attending a 20-h course for cheese evaluation (description and quantification of aroma, taste and texture). The sensory evaluation involved the use of two different tests: a discrimination test (paired comparison) and a descriptive test (quantitative descriptive analysis, QDA). The panel activity started with two attribute-generation sessions performed on commercial samples of PDO Canestrato Pugliese resulting in the definition of 13 attributes (5 regarding aroma, 4 taste and 4 texture). Then, the paired comparison was performed by offering the cheese samples in white plastic dishes identified by a 4-digit code in all possible pairwise options, with balanced presentation. The panelists were asked to assess the presence of a difference between the sample pairs and, if present, to indicate on a scorecard which sample had higher overall flavor intensity. Successively, the assessors performed the QDA analysis by tasting the samples one by one and judging them on a form in which the 13 established attributes were quantified on a 6-point scale. After statistical elaboration, the results of the analyses were discussed in a final open session.

### 2.5. Statistical Analysis

The data were statistically processed by XLSTAT software (version 2020.1.3, Addinsoft Inc., New York, NY, USA). Discrete variables were described by their mode values and continuous variables by their means. For microbiological analyses performed on milk, standard deviation (SD) was calculated. For chemical and microbiological analyses performed on cheese, the results were subjected to one-way ANOVA followed by Tukey’s honestly significant difference test at a critical value for significance of *p* < 0.05; as regards the sensory analysis, the nonparametric variables were compared by using the Kruskal–Wallis test. 

## 3. Results and Discussion

### 3.1. Chemical and Microbiological Analyses

**Figure 3:**
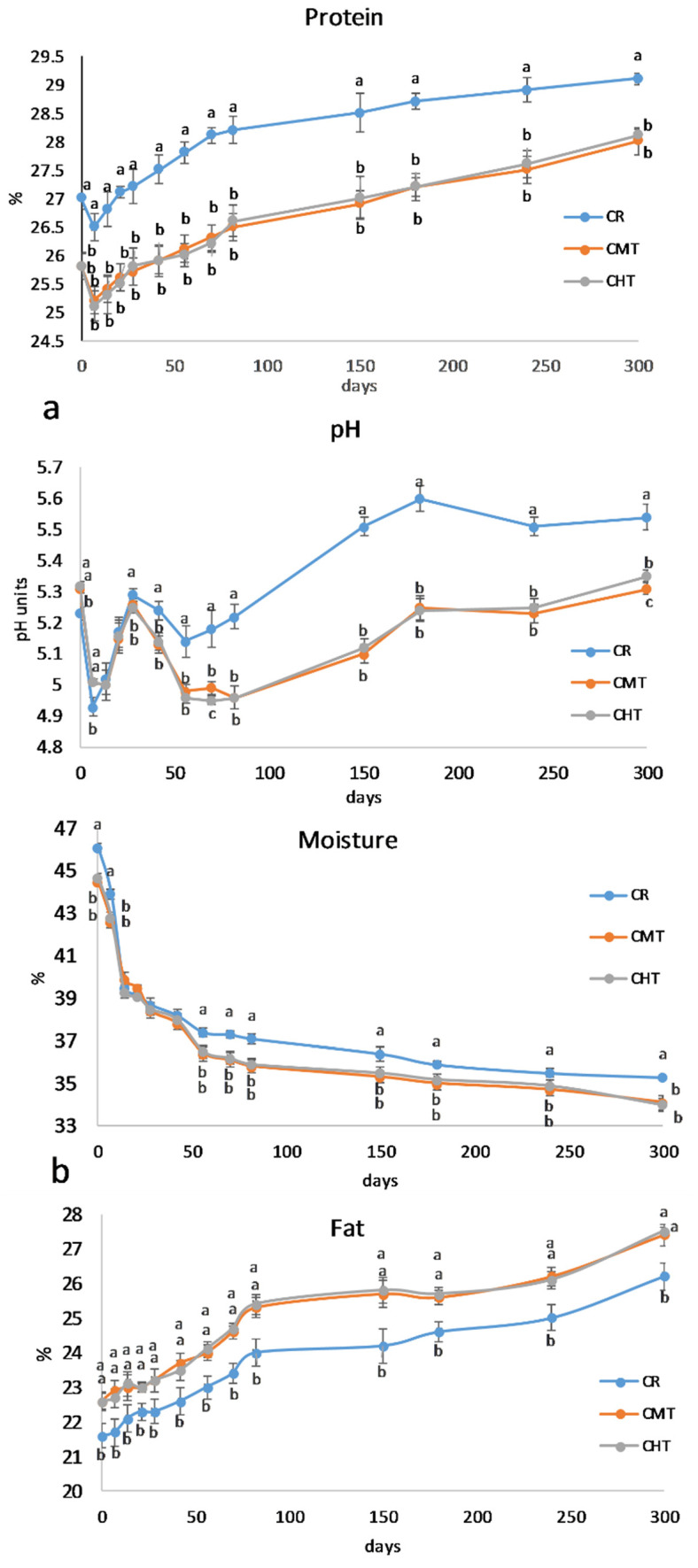
(**a**) Evolution of the moisture and fat contents in Canestrato Pugliese cheeses made from raw (CR), mild-thermized (CMT) and high-thermized (CHT) milk during ripening (0–300 days). Mean values of three analyses performed on two cheese wheels for each type of cheese. Different letters for the same ripening time indicate different values at *p* > 0.05; no letters means no difference. (**b**) Evolution of the protein content and pH in Canestrato Pugliese cheeses made from raw (CR), mild-thermized (CMT) and high-thermized (CHT) milk during ripening (0–300 days). Mean values of three analyses performed on two cheese wheels for each type of cheese. Different letters for the same ripening time indicate different values at *p* > 0.05.

The milk used to produce the cheeses contained 7.11% fat, 5.61% protein, and 4.64% lactose and had a pH of 6.55; somatic cell and total bacteria counts were 730,000 mL^−1^ and 540,000 mL^−1^, respectively. Thermization had a slight but significant effect on the cheese gross composition (Figure 3a,b). At the end of ripening, CMT and CHT were less humid and contained less protein and more fat than CR. On the other hand, the NaCl concentration was almost the same, ranging from a minimum of 3.10% in CMT to a maximum of 3.22% in CR. Overall, the compositional differences between raw and thermized cheeses were rather small, and were almost absent in the two thermized cheeses. It is worth highlighting that thermization had a scarce impact on moisture retention, differently from pasteurization that causes more water retention because of the greater denaturation of whey proteins, part of which tends to be entrapped into the curd [26,27]. 

In comparison with the compositional parameters, the differences in pH were much more relevant (see Figure 3b). Except for the samples analyzed at 7 days, the values were always lower in the thermized samples than in the raw one. The pH curves presented two “acidification peaks”, the first one after 7 days and the second one after 56 days. The first peak corresponded to the acidification phase of the starter, with the three samples behaving very similarly. The second acidification peak should be ascribable to other fermenting bacteria groups since the starters are known to undergo autolysis after the first stages of ripening [28]. In this phase, CR showed a significantly higher pH than CMT and CHT, which could be explained by the higher formation of low molecular weight alkaline compounds deriving from secondary proteolysis. This aspect will be further discussed later. 

Useful information about this point derived from the microbiological analysis of the vat milks (i.e., after the addition of the starter) and of the curds, whose results are shown in Table 2. As expected, the raw milk had higher counts than the two thermized milks, with mesophilic lactobacilli (MLb) and mesophilic lactococci (MLc) being the most represented groups, followed by enterococci (Ec). The same three groups were the most abundant in the two thermized milks, with the difference that Ec exceeded the other two. Enterococci are non-starter lactic acid bacteria that play an important role during ripening of artisanal hard and semi-hard cheeses [29]. According to Mc Auley et al. [30], their heat resistances greatly vary, depending on the species, and thermoduric enterococci can survive pasteurization. For TLb and TSt, which included the LAB species added with the starter, the higher counts in the raw milk indicated the presence of indigenous species that combined with the starter. It is worth mentioning that these two groups were not present at the level expected: that is not surprising, considering that the starter was added as a lyophilized culture, which needs time to grow, and that the milk samples were taken from the vat a few minutes after inoculation. The count value of total coliforms (TC) in the raw milk was rather high (5.47 cfu/g) but after thermization decreased by about 1.5–2.0 log units. Coliforms are commonly considered as an index of milk hygiene and their presence depends on the technological level of the farms and the efficiency of refrigeration throughout the whole production chain. In sheep milk, the populations are commonly higher than in cow milk: the survey conducted by de Garnica et al. [31] reported count values ranging from a minimum of 1.30 to a maximum of 6.64 cfu/g. The high TC count in the raw milk used in the present experimentation probably depended on the fact that, at the moment of the cheesemaking trials, the bulk milk used was the sum of 17 milk sub-batches having different quality and different levels of freshness (from a minimum of 18 h to a maximum of about 70 h). Indeed, that is the normal situation occurring in this geographical area, where the farms are scattered along a long route and, considering the low milk yield of sheep, milk collection takes a long time. 

As expected, the microbiological profile deeply changed at the end of the in-vat cheesemaking process, with a marked increase in all microbial groups in the curd. In particular, the differences in the LAB counts observed in the milks tended to disappear or decrease, whereas those regarding the other microbial groups remained relevant.

**Figure 4:**
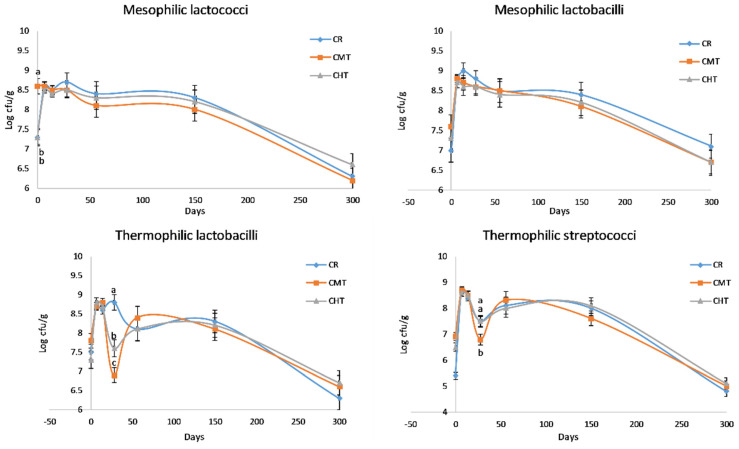
Counts * of the major lactic acid bacteria groups (cfu/g) in Canestrato Pugliese cheeses made from raw (CR), mild-thermized (CMT) and high-thermized (CHT) milk during ripening (0–300 days). Mean values of two analyses on two cheese wheels for each type of cheese.

**Figure 5:**
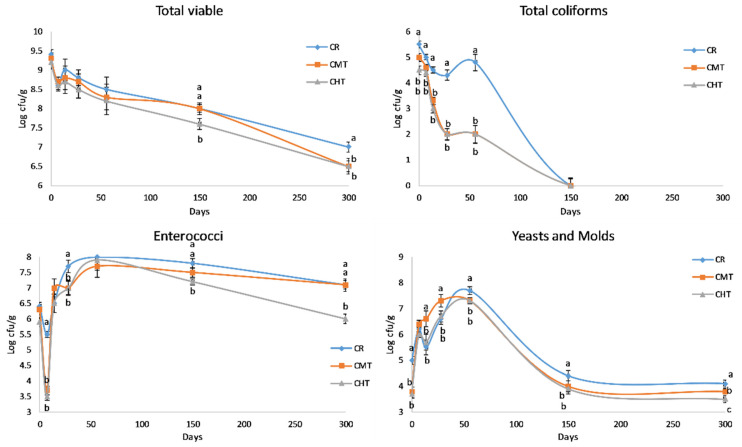
Counts of the non- lactic acid bacteria groups (cfu/g) in Canestrato Pugliese cheeses made from raw (CR), mild-thermized (CMT) and high-thermized (CHT) milk during ripening (0–300 days). Mean values of two analyses on two cheese wheels for each type of cheese.

Figure 4 shows the evolution of LAB counts in the cheeses throughout the entire ripening process. The curves of MLc and MLb were rather similar, with differences among the three cheeses corresponding at most to half a log unit. In the first weeks, these microorganisms reached a maximum count value of between 8.4 and 9.00 log cfu/g, and then constantly decreased over time. Albenzio et al. [20] reported the same trend for MLb in Canestrato Pugliese manufactured from raw and pasteurized milk, but the difference in the cell density between two cheeses was much higher with respect to our experimentation. This finding confirms that milk thermization allows for the preservation of a part of the indigenous LAB, maintaining a linkage with the territory. Differently from mesophilic, the two thermophilic LAB groups evidenced a dramatic drop at 28 days, except for the raw milk cheese that evidenced the drop of TLb at 56 days. These drops should correspond to the autolysis phase of the starter cultures, which in the CR cheeses was probably delayed (or made less evident) due to the presence of viable indigenous species. As for the non-LAB groups (Figure 5), the most relevant differences regarded total coliforms. In particular, CR had higher counts than the two thermized cheeses for the whole time in which this bacteria group was present. It is known that coliforms in hard cheeses tend to disappear rapidly in connection with the decrease in moisture content and increase in NaCl concentration. 

From the figure, it can be observed that the value of <1 log cfu/g was reached after about 3 months in CMT and CHT and about 4 months in CR. Another interesting feature was the evolution of enterococci. After an early drop, much more evident in thermized cheeses, the populations sharply increased and reached a maximum around 2 months, successively they started to decrease again but remained at high levels at the end of ripening. Interestingly, the trend in CR and CMT was similar from 4 months onwards, with count values at 300 days about 1 log unit higher than in CHT.

Finally, yeasts and molds evidenced a peak growth in correspondence with the second month, with count values that exceeded 7 log units cfu/g in all cheeses, with a maximum of 7.7 in CR. These values are very high, more than those reported in the same cheese by Corbo et al. [32], in which the maximum level approached 6 log units cfu/g at 19 days of ripening. However, the comparison is not reliable, as the authors did not perform the investigation on real PDO Canestrato Pugliese, whose weight must be of 7 or 14 kg, but on mini cheeses weighing only 1 kg. Another factor could be the ripening environments, which are known to play a major role in the contamination and growth of these microorganisms. In our experimentation, the cheese ripened on wooden boards in a natural warehouse with controlled temperature and relative humidity; in this room, after 1 month, the rind was totally covered by a lay of mold that underwent the first washing at 56 days, in perfect correspondence with the count peak.

**Figure 6:**
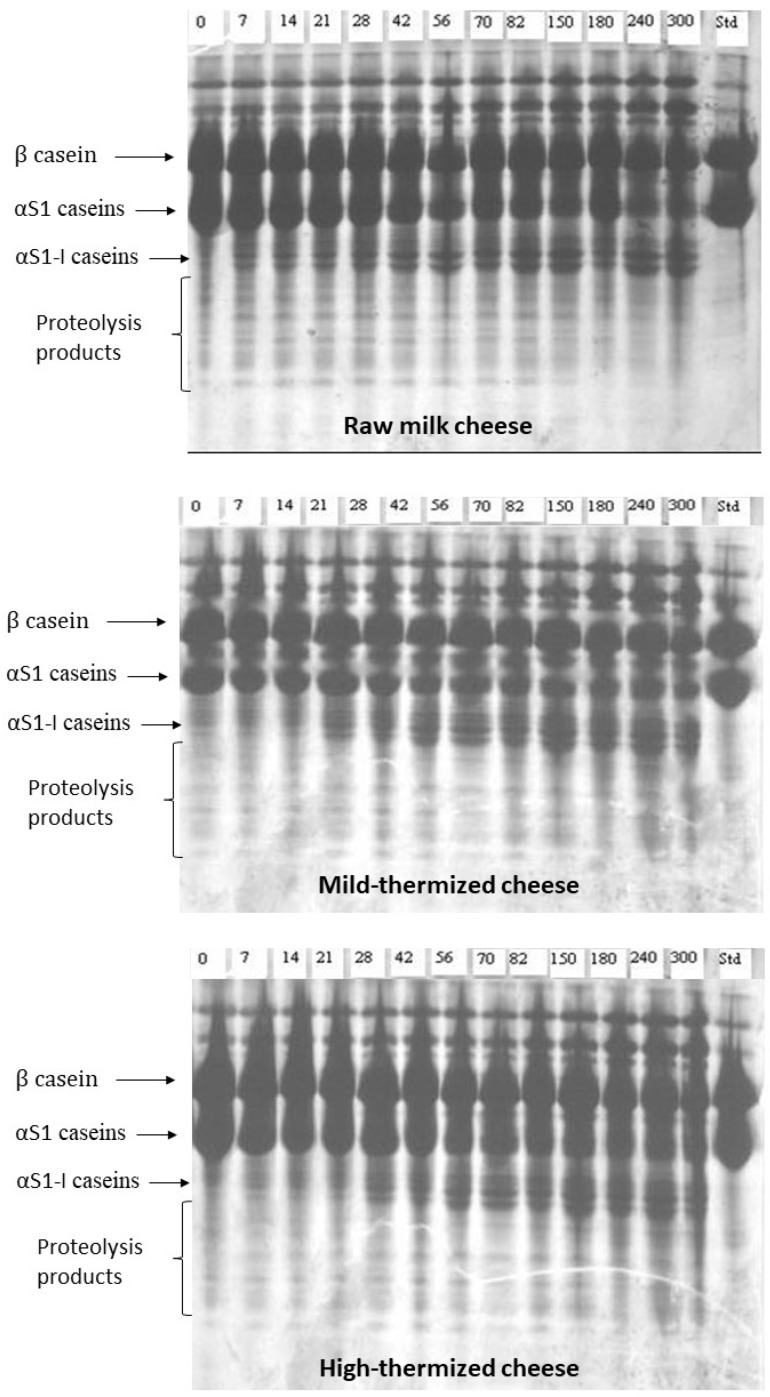
Urea-PAGE of Canestrato Pugliese cheese made from raw and thermized milk. Std = standard (sheep milk); from 0 to 300 = days of ripening.

Figure 6 shows the urea-PAGE electropherograms of the samples. The patterns were rather similar, indicating a relatively slow rate of proteolysis and faster degradation of α-S1 casein than β casein in all cheeses, in good agreement with the findings reported in a previous paper [33]. The main difference detectable between raw and thermized cheeses regarded the bands corresponding to the α-S1-I fraction and to the proteolytic products, which appeared to accumulate slightly faster in CR. This finding suggests a possible different rate of primary proteolysis but, considering that the electrophoretic techniques are semi-quantitative, this hypothesis needed confirmation. A further possible difference was observed in the zone between β and αS1 casein, where a diffuse unknown band is present. Even though the patterns in that zone suffer from smearing, it seems that this band was absent in milk and formed more intensely in the mild-thermized cheese. Pirisi et al. [34] reported a band positioned in the same area in Fiore Sardo cheese, and considered it as an unidentified compound, even though it was recognized by polyclonal antibodies against αS1-casein. In contrast, Sousa and Malcata reported a band in that area that was identified as a primary proteolytic product of β casein released by the activity of residual rennet [35]. 

**Figure 7:**
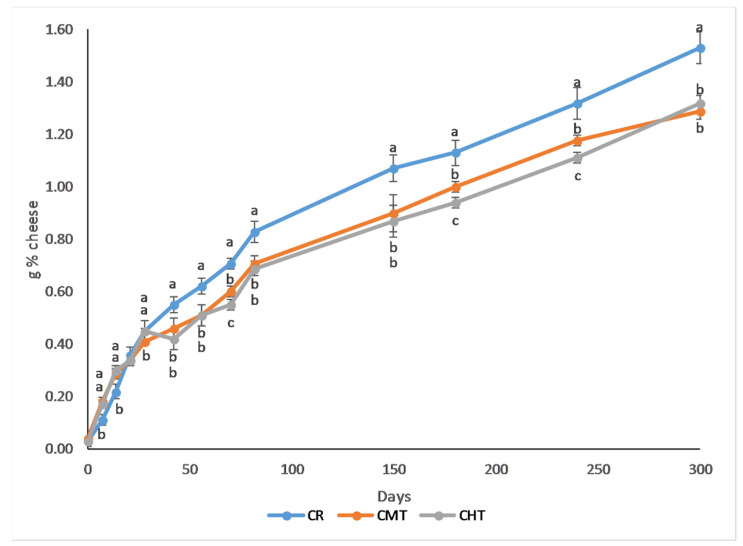
Evolution of the soluble nitrogen fraction in Canestrato Pugliese cheese made from raw and thermized milk. Different letters for the same ripening time indicate different values at *p* > 0.05; no letters means no difference.

Indeed, a confirmation was derived from the study of the soluble nitrogen fraction. In fact, the rate of its formation was almost the same until the first month of ripening; successively, it started to increase more rapidly in CR than in CMT and CHT and the final value in CR was about 20% higher (Figure 7). Consequently, the faster proteolysis in the raw milk cheese was confirmed. For a possible explanation, it must be considered that this biochemical event depends on the type of rennet used and the amount resituated in the curd, on the activity of the major milk protease (plasmin) and on the presence of proteolytic microorganisms [36]. In our experimental conditions: (i) the type of rennet was the same; (ii) the similar compositional characteristics of the curds should not have determined a different extent of rennet retention; (iii) the heat treatments applied were too weak to impact plasmin activity, since the whole plasmin system is affected only by higher time-temperature conditions [37]. Consequently, it is likely that the differences in proteolysis are attributable to the different activity of proteolytic microorganisms. As reported above, the raw milk cheese had higher counts of several potentially proteolytic microorganism groups (mesophilic lactic acid bacteria, enterococci, coliforms, and yeasts and molds) than the thermized ones. Given the high counts level observed for enterococci and their remarkable proteolytic activity [38,39], these microorganisms might have played a primary role. Nevertheless, taken together, the contribution to the peptidasic activity from the other groups should not be neglected, since the counts at some stages of ripening were relevant, as reported above for yeasts and molds. 

**Figure 8:**
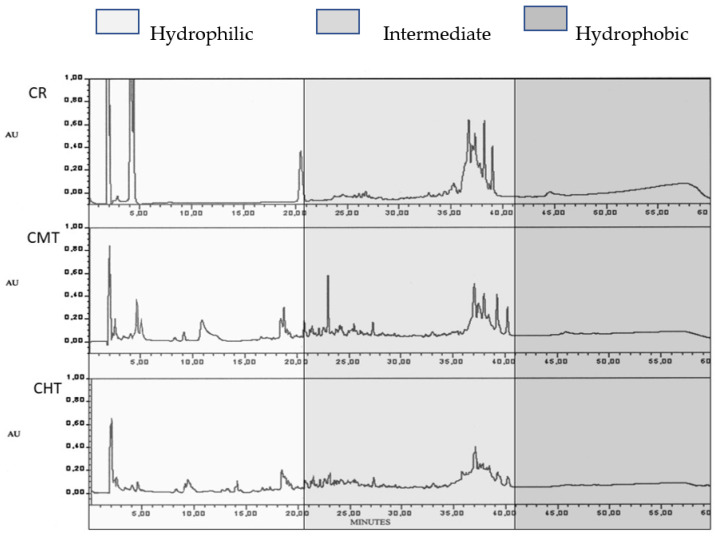
RP-HPLC of the soluble nitrogen fraction of Canestrato Pugliese cheese made from raw and thermized milk at 300 days of ripening. CR = raw milk cheese; CMT = mild-thermized cheese; CHT = high-thermized cheese.

The RP-HPLC study of WSN at 300 days of ripening revealed both quantitative and qualitative differences, supplying further information about proteolysis (Figure 8). The raw milk cheese had a higher total peak area and a less complex profile with respect to the other two cheeses, which were rather similar to each other. For a better interpretation of the qualitative differences, the chromatograms were divided into three parts: hydrophilic, intermediate and hydrophobic. It is known that the hydrophilic peaks mostly correspond to small nitrogen compounds with polar characteristics such as hydrophilic free amino acids and small peptides, resulting from the activity of bacterial peptidases [40,41]. In CR, the hydrophilic part was characterized by three major peaks (the first one corresponding to non-retained compounds), whose sum represented more than 60% of the total peak area; in CMT and CHT, a high number of peaks was present, with different retention times and a much lower area with respect to CR. The intermediate part of the chromatogram also showed significant differences between raw and thermized cheeses, but in this zone, the differences were mostly quantitative. The huge total area of hydrophilic peaks in the raw milk cheese fitted well with the above hypothesis that proteolytic microorganisms were mainly responsible for faster proteolysis. 

### 3.2. Sensory Analysis

During the paired comparison tests, all panelists were able to discriminate the raw milk cheese from the thermized ones that, in turn, were not judged as different from each other. QDA analysis allowed for deepening the reasons for the differences perceived, as shown in Table 3. CR cheese received significantly higher scores for three aroma (cheesy-sweat, sheep barn and dirty socks), one taste (spicy) and one texture (eyes) attributes. Overall, during the final discussion, the panel defined it as “rough” and “typical”, whereas the terms used to define the two thermized cheeses were “fragrant” and “correct”. Information is available in the literature on the origin of the three aroma descriptors that discriminated the samples. The cheesy-sweat odor is mostly connected to short chain free fatty acids, such as butanoic and hexanoic [42,43]: the former can both derive from microbial fermentation and lipolysis, the latter only from lipolysis. Unfortunately, we did not investigate the volatile compounds, nor lipolysis. The sheep barn odor has been reported to have strong correlation with *p*-cresol, a phenolic compound directly deriving from milk and closely dependent on animal feeding, with milk from grass-fed animals presenting higher concentration with respect to milk from total mixed ratio-fed animals [44]. As in the present investigation, the milk used was the same in all cheesemaking trials, so this sensory attribute should have a different origin. According to the literature, *p*-cresol in cheese can also derive from the microbial catabolism of tyrosine [45]. The volatile compound responsible for the dirty socks odor in cheese (3-methylbutanoic acid) also derives from the microbial catabolism of an amino acid (leucine) [46,47]. In the present experimentation, the higher secondary proteolysis observed in the raw milk cheese in connection with the higher counts of proteolytic microorganisms, could fit these pathways. Finally, the higher scores for the spicy and eyes descriptors can also be attributed to the different microbiological profile. In hard cheese, the spicy taste is a typical consequence of advanced lipolysis with the formation of short-chain free fatty acids, mostly butanoic and hexanoic, the same that are responsible of the cheesy-sweat odor. Even though we did not investigated lipolysis, it is worth mentioning that many strains belonging to both coliforms and enterococci groups can be strongly lipolytic [48]. The higher coliform counts can also be a good candidate to explain the presence of small eyes in the cheese, which were already evident after a few weeks of ripening. 

## 4. Conclusions

The application of milk thermization in the manufacturing process of Canestrato Pugliese PDO cheese affected the overall quality. Even though the gross composition underwent only minor changes, the sensory characteristics of the raw milk cheese were more intense with respect to the thermized ones, despite of the use of the same starter in the manufacturing process. The result of the investigation clearly addresses a relevant role of the indigenous microbiota, which is probably responsible of faster secondary proteolysis with the related aroma-active molecules. The most important information that can be drawn from the present work is that heat treatment alone cannot be proposed as a tool to minimize defective productions, since the original characteristics of the cheese tend to fade. The only possible solution might be the use of an autochthonous whey starter, able to maintain the linkage with the territory and provide a complex microbiota, resembling the main microbiological characteristics of raw milk as much as possible, including the presence of enterococci. Although they are not yet considered GRAS (generally recognized as safe) microorganisms, their presence in a long-ripened hard cheese such as Canestrato Pugliese does not raise safety concerns. 

## Figures and Tables

**Table 1:** PDO and PGI Italian cheeses made from sheep milk (alone or in combination) and heat treatments allowed.

Cheese	Type of Milk	Acknowledgement	Treatment Allowed
1. Canestrato di Moliterno	sheep + goat	PGI	thermization
2. Canestrato Pugliese	sheep	PDO	none
3. Casciotta d’Urbino	sheep + cow	PDO	pasteurization
4. Fiore Sardo	sheep	PDO	none
5. Formaggio di Fossa di Sogliano	cow + sheep	PDO	pasteurization
6. Murazzano	sheep + cow	PDO	any heat treatment
7. Pecorino Crotonese	sheep	PDO	any heat treatment
8. Pecorino delle Balze Volterrane	sheep	PDO	none
9. Pecorino del Monte Poro	sheep	PDO	none
10. Pecorino di Filiano	sheep	PDO	none
11. Pecorino di Picinisco	sheep	PDO	none
12. Pecorino Romano	sheep	PDO	thermization
13. Pecorino Sardo	sheep	PDO	any heat treatment
14. Pecorino Siciliano	sheep	PDO	none
15. Pecorino Toscano	sheep	PDO	any heat treatment
16. Piacentinu Ennese	sheep	PDO	none
17. Vastedda del Belice	sheep	PDO	none

**Table 2:** Counts * of the major microorganism groups (cfu/g) in the vat milk (after starter addition) and in the curd. RM = raw milk; MTM = mild-thermized milk; HTM = high-thermized milk. TV = total viable; TC = total coliforms; Ec = enterococci; MLb = mesophilic lactobacilli; TLb = thermophilic lactobacilli; MLc = mesophilic lactococci; TSt = thermophilic streptococci; Y&M = yeasts and molds. Mean values ± standard deviation from two analyses on each type of milk and curd.

Group	RM	MTM	HTM	Curd R	Curd MT	Curd HT
TV	6.69 ± 0.42	5.17 ± 0.34	5.85 ± 0.21	9.17 ± 0.34	8.87 ± 0.21	8.91 ± 0.27
TC	5.53 ± 0.34	3.93 ± 0.04	2.55 ± 0.15	5.47 ± 0.24	4.05 ± 0.03	3.40 ± 0.14
Ec	5.89 ± 0.29	4.82 ± 0.11	4.40 ± 0.64	6.28 ± 0.11	5.35 ± 0.10	4.57 ± 0.15
MLb	6.67 ± 0.34	4.68 ± 0.28	4.26 ± 0.21	7.05 ± 0.09	5.98 ± 0.25	5.36 ± 0.12
TLb	5.32 ± 0.09	3.41 ± 0.31	3.63 ± 0.22	7.28 ± 0.24	6.15 ± 0.19	6.28 ± 0.04
MLc	6.21 ± 0.30	4.30 ± 0.43	3.52 ± 0.22	6.91 ± 0.18	6.00 ± 0.08	5.82 ± 0.25
TSt	2.71 ± 0.20	1.57 ± 0.11	2.09 ± 0.15	5.49 ± 0.11	5.56 ± 0.15	5.51 ± 0.06
Y&M	5.40 ± 0.08	3.53 ± 0.05	2.07 ± 0.56	5.40 ± 0.08	3.77 ± 0.10	3.55 ± 0.20

**Table 3:** Sensory attributes (modal values) for Canestrato cheese made from raw and thermized milk at 300 days of ripening. CR = raw milk cheese; CMT = mild-thermized cheese; CHT = high-thermized cheese. Sig = statistical significance (* = different at *p* < 0.05).

Attributes	CR	CMT	CHT	Min–Max	Sig
AROMA					
Cheesy-sweat	4	3	3	3–5	*
Sheep barn	2	0	0	0–2	*
Butter	1	1	1	0–1	
Toasted	1	1	1	0–1	
Dirty socks	2	0	0	0–3	*
TASTE					
Salty	2	2	2	2–3	
Bitter	0	0	1	0–1	
Umami	2	2	2	1–2	
Spicy	2	1	1	1–2	*
TEXTURE					
Eyes	2	1	0	0–2	*
Hard	4	4	4	3–4	
Crumbly	3	3	3	2–3	
Greasy	3	3	3	2–3	
Soluble	3	2	2	2–3	

## Data Availability

Data is contained within the article.
